# Determination of Methomyl Residues in Bohe by Ultrahigh-Performance Liquid Chromatography-Tandem Mass Spectrometry (UPLC-MS)

**DOI:** 10.1155/2020/8817964

**Published:** 2020-11-05

**Authors:** Yao Zheng, Addotey Tracy Naa Adoley, Benkhelifa Fateh, Wei Wu, Gengdong Hu, Liping Qiu, Jiazhang Chen

**Affiliations:** ^1^Freshwater Fisheries Research Center, Chinese Academy of Fishery Sciences, Fishery Eco-Evironment Monitoring Center of Lower Reaches of Yangtze River, Laboratory of Quality & Safety Risk Assessment for Aquatic Products on Environmental Factors (Wuxi), Ministry of Agriculture, Wuxi, Jiangsu 214081, China; ^2^Wuxi Fishery College, Nanjing Agricultural University, Wuxi, Jiangsu 214081, China

## Abstract

The aim of this work is to investigate the presence of methomyl pesticide residue and the rate of disappearance in mint cultivated in the aquaponics system based on the application of UPLC-MS to establish a safety time interval before crop harvesting. Results showed that an effective and sensitive method based on UPLC-MS has been used for the determination of methomyl pesticide residues in mint. The initial residue level was much higher in roots (79.52 *μ*g/kg), and it can be decreased to 16.73 (after 15 days) *μ*g/kg and 3.31 (20 days) *μ*g/kg, while the least was detected on the mix leaves and stems (44.54 *μ*g/kg), and it can be decreased to 15.35 (after 20 days). In our case, we suggest that a safety interval in the range of 15–20 days should be allowed after the detection of methomyl in water, and the concentration of methomyl was lower than the acceptable daily intake (ADI) of the China Food and Drug Administration (CFDA) (20 *μ*g/kg).

## 1. Introduction

The insecticide methomyl (S-methyl N-methylcarbamoyloxy thioacetimidate, CAS 16752-77-5) is an oxime carbamate insecticide applied to control a wide range of insect classes [1], in order to increase crop production and farming profits. However, methomyl was classified by the US EPA (Environmental Protection Agency) as a Class I restricted-use pesticide (RUP). Considered as one of the highly toxic pesticides, methomyl is introduced into aquatic systems by agricultural activities [2]. Although methomyl has been widely used to treat field crops and has high water solubility, it has only infrequently been detected as a contaminant of water bodies. Previous studies were conducted on the effect of methomyl pesticides for several fish such as the acute toxicity of methomyl on the tilapia [3].

Mint (*Mentha haplocalyx*) or bohe in Chinese is an herbaceous plant that originates from the Mediterranean region and is cultivated worldwide. Mint is widely consumed as herbal teas and is one of the popular herbs used in traditional medicine and ethno medicine against a large variety of diseases and an important material for culinary purposes and fragrance in cosmetics [4]. Moreover, mint is a suitable herb for integrated aquaculture-agriculture farming and aquaponics systems [5]. The usage of menthol, the active compound in mint, was 35000–40000 t all over the world and 3500–3800 t in China.

Pesticide residue analysis can be considered as a difficult task since large amount of chlorophylls, oil, and other colored compounds are contained in mint. Sample preparation is a critical step because of the complex matrixes. The procedures of extraction and cleanup are often crucial steps that ameliorate the analytical determination speed and sensitivity and facilitate identification and quantification of low concentration of pesticide. In this study, various methods have been developed, such as stir bar sorptive extraction (SBSE), pressurized liquid extraction (PLE), solid-phase microextraction (SPME), matrix solid-phase dispersion (MSPD) solid-liquid extraction (SLE), and solid-phase extraction (SPE) [6].

SPE was the one that has been commonly applied for pesticide residue analysis. In this work, a Cleanert TPT cartridge was used to clean up the mint sample. Zhao et al. [7] reported that Cleanert TPT cartridge resulted in great improvement when compared to other SPE cartridges since it has been made of amide-modified polystyrene, graphitized carbon black, and polyamine silica [8].

Several methods have been reported to determine methomyl and other pesticides residue in mint using gas chromatography [9, 10], liquid chromatography [11], gas-liquid chromatography [12], high-performance liquid chromatography (HPLC), high-performance liquid chromatography-mass spectrometry (HPLC-MS), and HPLC-MS/MS. On the other hand, ultrahigh-performance liquid chromatography (UPLC) presents an interesting alternative for methomyl pesticide residue determination in traditional Chinese medical herbs [6, 13], a method which is an advanced form of HPLC with improved solution, enhanced sensitivity, and shorter retention times [14].

To the best of our knowledge, only one scientific report has been reported on the presence of methomyl pesticide residue and rate of disappearance in peppermint after foliar application [9]. The aim of this work is to investigate the presence of methomyl pesticide residue and the rate of disappearance in mint cultivated in the aquaponics system based on the application of UPLC-MS to establish a safety time interval before crop harvesting.

## 2. Materials and Methods

### 2.1. Chemicals and Reagents

This study was conducted in the Freshwater Fisheries Research Center, Chinese Academy of Fishery Sciences, China. The mint samples from floating bed [15, 16], made using PVC pipes (Φ 40 mm, 2 m*∗*2 m as a template frame, combined all frames with the strings) and nylon mesh (6 strands, Φ 10 mm), were collected from the aquaponics system, using genetically improved farmed tilapia (GIFT, *Oreochromis niloticus*) as a model for fish culture, and the concentration of methomyl (C_5_H_10_N_2_O_2_S, Shanghai Focus Biological Technology Co., Ltd, China) added to the water was 200 *μ*g/l. Temperatures for weather and water in fish tanks (40 cm × 40 cm × 60 cm, and 100 L) were recorded during the experiment. Minimum and maximum temperatures varied between 25–31°C and 27–30°C, respectively.

Collected samples were soaked in water for 5 min, rinsed under running water for 2–3 min, and then separated into two groups; the first one was mint roots, and the second were mint leaves and stems. All samples were oven-dried for 6 h at 60°C and finally powdered. The modified methods of Díaz-Maroto et al. [17] were used in this study.

Mint samples (2.5 g) were placed in a 50 mL centrifuge tube, mixed with 15 mL acetonitrile and homogenized for 5 min at 500 rpm. The mixture was centrifuged at 8,000 rpm for 5 min. Supernatants were transferred to a flask; the same extraction procedure for supernatant was repeated. Using a rotary evaporator at 40°C, the resulted supernatants were concentrated to about 1 mL for the SPE cleanup.

### 2.2. UPLC-MS Conditions

Chromatographic analysis was performed using a Waters Acquity UPLC system combined with a Xevo^TM^ TQ mass spectrometer (Waters, USA). Data acquisition and processing were performed using Masslynx 4.1 software. In this study, chromatographic separations were attained using Waters Acquity UPLC BEH C18 column (2.1 mm × 100 mm, 1.7 *μ*m particle size) with a binary mobile phase. Gradient elution was achieved with 0.1% (*v/v*) formic acid in water as mobile phase *A* and acetonitrile as mobile phase *B*. Both solutions of the mobile phase were pumped at a flow rate of 0.3 mL/min. The injected sample volume was kept at 5 *μ*L. The temperature of the column was maintained at 30°C, and the sample managing temperature was at 4°C.

The gradient program (Table S1) was started with 90% component *A* (10% *B*) at injection time and inversed to 10% component *A* (90% *B*) in 2.5 min, further to 50% *A* (50%) over 3 min and increased linearly to the initial starting condition, and kept there for 1.5 min.

Mass spectrometric analysis was achieved using multiple reaction monitoring (MRM) operating in both positive and negative ion modes. The ESI ion source parameters were as follows: capillary voltage, 3.5 kV; extractor voltage, 30 V; source temperature, 450°C; desolvation temperature, 450°C; desolvation gas (nitrogen) flow, 800 L/h; cone gas (nitrogen) flow, 50 L/h.

### 2.3. Samples Pretreatment and Cleanup

The Cleanert TPT cartridge is a novel mixed multilayer SPE mode cartridge composed of three different layers that are specially designed for plant cleanup, which has advantages of operational simplicity, rapidity, and high recovery cartridge [18]. It has been widely used in tea samples from the European Union and Japan, which had a well recovery rate, RSD, determination coefficient (*R*^2^), LOQs, and LOD [18–20]. Na_2_SO_4_ (2 gm) was transferred into the Cleanert TPT cartridge (1,000 mg, 06 mL; Agela, China). To activate the cartridge, 0.5 mL acetonitrile-toluene (3 : 1, *v/v*) was used. The residue in the evaporation flask was dissolved by 5 mL acetonitrile-toluene (3 : 1, *v/v*) three times, and the final solution was added to the cartridge. 25 mL of acetonitrile-toluene (3 : 1, *v/v*) was used to elute the cartridge. The eluent was collected with a rotary evaporator and evaporated to dryness at 40°C. The dried residue was washed with 1.5 mL acetonitrile and filtered with an organic membrane (0.22 *μ*m) for UPLC-MS analysis.

To evaluate the linearity of the calibration curve, the standard stock mixture solutions were prepared in pure methanol and blank mint sample at six concentration levels (1, 5, 10, 20, 50, and 100 *μ*g/L); the linearity equation was *Y* = 951.1 x + 1528.6 (*R*^*2*^ = 0.9938).

## 3. Results and Discussion

In this study, methomyl residues in mint cultured in the aquaponic system with a known concentration (200 *μ*g/L) of the target pesticides in water were investigated. Results are presented in Table 1. The initial residue level was much higher that that in roots, presented as 79.52 *μ*g/kg, while the least was detected in the mix samples of both leaves and stems, presented as 44.54 *μ*g/kg. The residue levels decreased steadily over the 20-day study period. A similar trend of results has been explained in peppermint and spearmint after foliar and ground application [9] and also in strawberry, tomato and cucumber [21].

The amount of residues was found to be lower than half in 15 days when compared with time zero sample. Foremost, methomyl aqueous solutions have been reported to decompose faster on aeration and sunlight. In similar condition of our experiment, the aqueous half-life estimated for methomyl in fish tanks is 6 days.

In turn, this decline was not independent of environmental and study conditions [22]. Therefore, temperature was a key factor for pesticides dissipation from plants [23]. In our case, the experiment was conducted in summer and the temperature was relatively high which increases volatilization from the plant surface to air. This process was identified by Wolters et al. [24], as one of the main operations determining dispersion throughout the environment.

Mint has an exponential growth phase from June to September. Growth is a well-known dissipation process that does not reduce compounds' mass but leads to lower concentration by dilution. Furthermore, growth can be a very effective dissipation process when pesticides are applied during the blooming growth phase [25]. Miles et al. [26] estimated that growth dilution had the same importance for dissipation as volatilization.

In the same context, degradation has been stated to be one of the principal dissipation processes [27]. Harvey and Reiser [28] investigated the metabolic fate of methomyl in tobacco, corn, and cabbage. Plants treated with radiolabeled methomyl rapidly degraded the compound to carbon dioxide and acetonitrile, which volatilized from the plant tissues. After whole decomposition of the methomyl molecule, the remainder of the I4C activity had been reincorporated into natural plant components.

## 4. Conclusion

In summary, an effective and sensitive method based on UPLC-MS has been used for the determination of methomyl pesticide residues in mint. The results of measured residues in leaves/stems and roots showed reasonable dissipation. This can be explained by the volatilization, growth dilution, and degradation. In tune with the China Food and Drug Administration (CFDA), the present preliminary results showed that the recorded maximum residue was inferior to the maximum residue limit (200 *μ*g/kg) released by the National Food Safety in Standard Maximum Residue Limits for Pesticides in Food (GB 2763–2016). Moreover, CFDA estimated the acceptable daily intake (ADI) of methomyl, without generating detectable health hazards below 20 *μ*g/kg. In our case, we suggest that a safety interval in the range of 15–20 days should be allowed after the detection of methomyl in water. However, further statistical analysis using a larger cohort of samples would need to be carried out in order to fully validate this conclusion.

## Figures and Tables

**Table 1:** Methomyl residues in mint cultured in the aquaponic system with known concentration (200 *μ*g/L).

Time (days)	Methomyl residue in mint			
	Hay		Roots	
	Mean residue (*μ*g/kg) (±SE)	Reduction (%)	Mean residue (*μ*g/kg) (±SE)	Reduction (%)
0	44.54 ± 0.56	—	79.52 ± 0.30	—
5	39.29 ± 0.23	11.79	68.12 ± 0.93	14.34
10	30.07 ± 0.41	23.47	47.61 ± 0.67	30.11
15	16.73 ± 0.25	44.36	25.29 ± 0.35	46.88
20	3.310 ± 0.20	80.22	15.35 ± 1.02	39.30

## Data Availability

The data are available when published.

## Supplementary Materials

Supplementary Materials Table S1: flow phase and gradient elution conditions.
