# Supplementary material for: Determination of Methomyl Residues in Bohe by Ultrahigh-Performance Liquid Chromatography-Tandem Mass Spectrometry (UPLC-MS)
Source: Int J Anal Chem. 2020 Nov 5;2020:8817964. doi: 10.1155/2020/8817964 (PMC7661117; doi:10.1155/2020/8817964)
Supplement: Supplementary Materials — Table S1: flow phase and gradient elution conditions. [file 8817964.f1.doc]

**Table S1** Flow phase and gradient elution conditions.

| Time/min | Flow/(mL·min^-1^) | A% | B% | Curve |
| --- | --- | --- | --- | --- |
| Initial | 0.3 | 90 | 10 | Initial |
| 2.5 | 0.3 | 10 | 90 | 3 |
| 3 | 0.3 | 50 | 50 | 6 |
| 3.5 | 0.3 | 90 | 10 | 6 |
| 5 | 0.3 | 90 | 10 | 6 |
| A: 0.1% formic acid water; B: acetonitrile | | | | |
